# Inclusive mentorship of pediatric trainees: pediatric oncology as a microcosm

**DOI:** 10.3389/fonc.2025.1531784

**Published:** 2025-01-31

**Authors:** Sadhana Jackson, Jessica W. Tsai, Kyle L. MacQuarrie

**Affiliations:** ^1^ Surgical Neurology Branch, National Institute of Neurological Disorders and Stroke (NINDS), Bethesda, MD, United States; ^2^ Pediatric Oncology Branch, National Cancer Institute (NCI), National Institutes of Health, Bethesda, MD, United States; ^3^ Children’s Hospital Los Angeles, Cancer and Blood Disease Institute, Los Angeles, CA, United States; ^4^ Department of Pediatrics, Keck School of Medicine, University of Southern California, Los Angeles, CA, United States; ^5^ Stanley Manne Children’s Research Institute, Ann and Robert H. Lurie Children’s Hospital of Chicago, Chicago, IL, United States; ^6^ Department of Pediatrics, Northwestern University Feinberg School of Medicine, Chicago, IL, United States

**Keywords:** diversity, equity, inclusivity, mentorship, pediatric oncology

## Abstract

Mentorship is a critical part of career development for medical professionals. Mentees find value in mentors who share parts of their identity, and this role-modeling improves career development. In pediatric hematology-oncology specifically – reflective of academic medicine more broadly - the current pool of mentors is less diverse than the pool of mentees. Mentoring consciously in an inclusive manner is a way to support all mentees, not just those who share identity with the mentor. Utilizing skills such as microintervention and bystander intervention, all while focusing on allyship are tools that mentors can develop and use to improve their mentoring practices.

## Introduction

Mentorship is a critical component of the development of medical and scientific professionals. Supportive academic mentors provide hands-on patient care, scientific expertise, and advice to their mentees, and serve as role models on which trainees can base their goals for personal and professional success. Mentees find value in role model mentors who share components of their identity in developing their own career and scientific identity (1, 2), and this development is linked to increased self-efficacy and improved career development over time (3–5).

Regardless of whether a mentor and mentee share like components of their identities, all mentees can benefit from inclusive mentorship. Inclusiveness in the setting of mentorship refers to multiple concepts – not only ‘embracing’ differences between mentors and mentees and amongst mentees, but working to ensure that mentees feel welcomed, valued, and supported (6). Recognizing the value of a mentee’s experiences forms the foundation of inclusive mentoring (7); in turn, mentoring that ignores or detracts from a mentee’s identity can be seen as detrimental. The potential for a negative impact on mentee well-being has worsened recently in the United States with backlash against the value of diversity, equity and inclusion (DEI) efforts. In addition, multiple US Supreme Court decisions in the past few years have infringed on human rights and ideals, with ‘303 Creative LLC v. Elenis’ ruling that a Colorado business owner could refuse service to LGBT+ customers due to her personal religious objections and ‘Dobbs v. Jackson Women’s Health Organization’ ending the constitutional right to abortions nationally. Those mentees who have had their rights threatened would benefit from mentoring that recognizes the effect on them and supports them appropriately. Given the prevalence of heterosexual white males in the pool of medical and scientific physician mentors, we posit that those mentees who do not share that same identity are particularly at risk of feeling unsupported. This in turn could risk worsening the already existent disparities in job dissatisfaction and attrition of trainee physicians from underrepresented and marginalized groups (8–11).

Historically, the workforce of the discipline of pediatric hematology/oncology (PHO) has been a microcosm of the broader medical and STEM communities in the United States. In 2021, 64% of the US national STEM workforce was white, with a nearly identical percentage identified as male (12). In 2018, 64.1% of practicing physicians identified as male, and while fewer physicians identified as white (56.2%), medical school faculty were less diverse, with 63.9% identifying as white (13). In 2015, the PHO physician workforce had 59% identifying as male, and 78% as white (14, 15). In the last decade, a significant and ongoing change has been seen in workforce characteristics. As of 2023, the proportion of male-identified faculty has decreased to 40%, and while the majority (64%) still identify as white, that represents a 14% decrement. This trend is even more notable in recent trainee data: fewer than a third of current trainees (31%) identify as male, and 56% as white (16). While the authors all practice in the US and are most familiar with those trends, it appears that similar forces are present elsewhere; 2024 data from the General Medical Council of the United Kingdom demonstrates a similar shift in practitioner gender. The workforce shifts from being male-identified predominant for those physicians over the age of 45 to female-identified predominant for physicians less than 45 years old. Despite this, female-identified physicians make up only 39% of the subspecialty physician workforce (17, 18).

In 2018, the Children’s Oncology Group (COG) Young Investigator Mentoring program – designed to train early-career pediatric oncologists - published mentorship statistics. While the significant majority of COG member institutions are located in the US, it includes institutions located in Canada, Australia, New Zealand, and Saudi Arabia as well. There was clear discordance between the population of mentors and mentees, with 60% percent of mentors identified as males, but only 39% of the mentees (19, 20). Absent from the program survey were statistics on race, ethnicity, sexual orientation, or gender identity. Another notable example of a general lack of data on identity is physician self-identification as gender non-binary; the American Board of Pediatrics didn’t even begin to collect such data until 2021. Acquisition of identity data on a routine basis will be critical to understanding the trajectory of the developing workforce.

If the trends seen in pediatric oncology over the past decade continue, and current mentees go on to become future mentors, then the identity disparity between mentors and mentees will decrease over time. Given the current characteristics of PHO trainees, it may seem a foregone conclusion that this process will occur, but both historically and currently, the relative proportion of male and white-identified medical school faculty members increases along with advancing academic rank (13) and so data collection will be necessary to confirm if that trend of increasing concordance between mentors and mentees actually occurs. And regardless of the trends of a shifting workforce, current – not just future - mentees deserve mentoring that consciously works to support them and all the facets of their identities.

**Figure 1:**
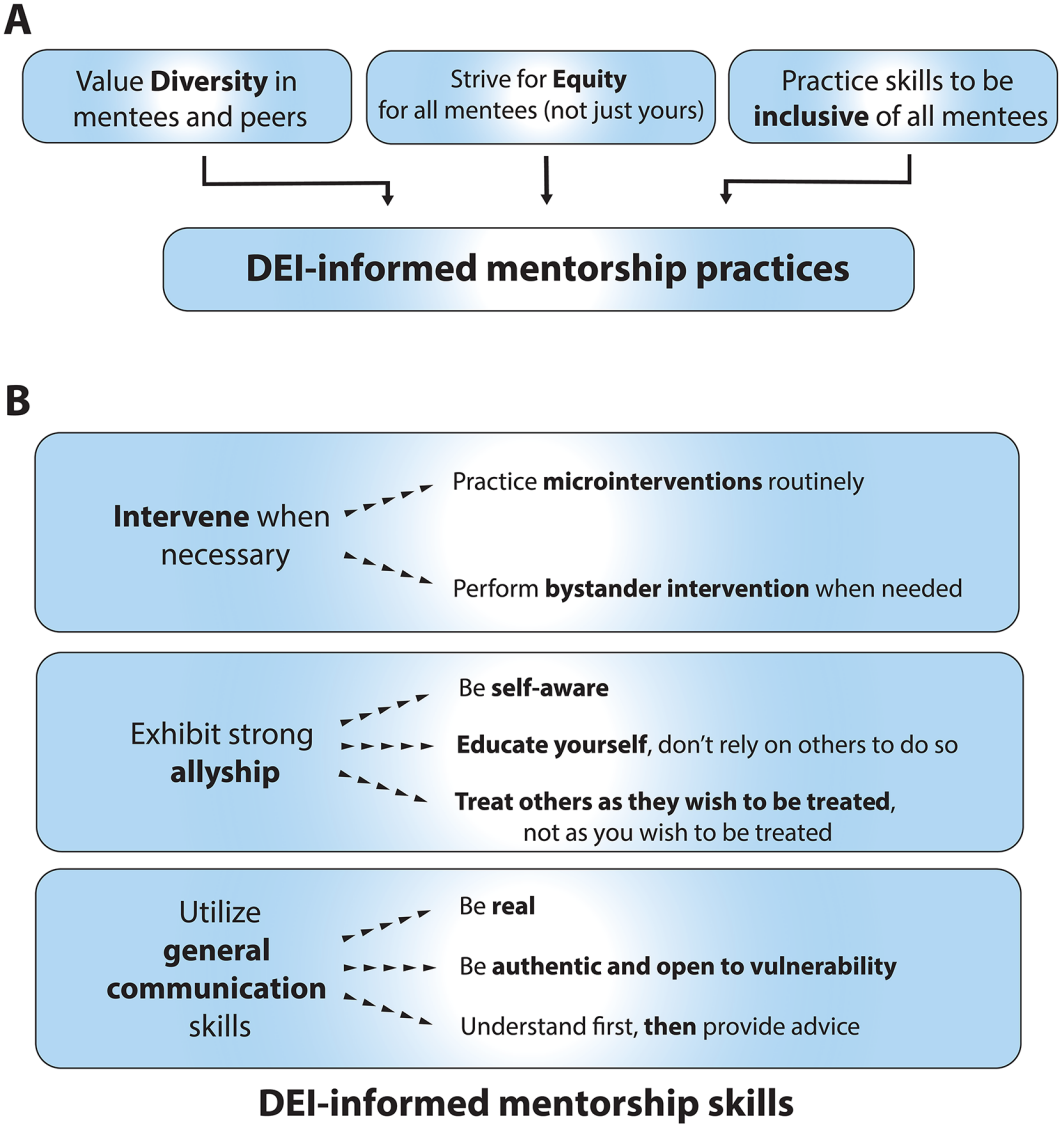
DEI-informed approaches to mentorship. **(A)** A diagrammatic depiction of how diversity, equity and inclusion can inform mentorship practices. **(B)** The mentorship skills of *intervention*, *allyship*, and *general communication strategies* are broken down into specific actions.

The positive and supportive role of mentorship and sponsorship is imperative not only to the success of the mentee, but also to improved clinical care. Previous studies have shown that homogeneity can negatively impact patients and is reflected in the lack of racial/ethnic diversity seen with clinical trial enrollment and equity of available clinical support services (21–23). Inclusive mentorship can therefore have positive long-term effects on career achievements, patient well-being and overall productivity within the field. Fortunately, the lessons and tools available more broadly for diversity, equity, inclusion and justice (DEIJ) efforts can also be utilized in the pediatric oncology mentoring space. Through the combination of valuing diversity, striving for equity, and continuing to work on being inclusive of all mentees (**Figure 1A**), mentorship can – first and foremost - better serve mentees. In addition, it can produce more ‘tangible’ benefits, including improved clinical outcomes, enhanced research creativity and productivity, and a positive quality of work life.

## Diversity, equity, inclusion, and justice impact the needs of mentees in numerous ways

### Diversity and inclusion

Aside from the moral imperative of mentors supporting diverse mentees, diversity itself has tangible benefits. Diversity in gender and ethnicity corresponds to better financial performance, improved employee satisfaction, and gains in the “war for talent” (24–26). Scientists from underrepresented groups produce work that is more ‘novel’ (27). The risk of losing diverse mentees and impeding productivity is a sobering reality. MD-PhD trainees show differences in leaving their training programs related to their ethnicity and race (8). The pathway for those underrepresented in surgical careers appears to be narrowing (28). Persons identifying as lesbian, gay, bisexual, transgender, and more (LGBT+) who do not disclose their identity, from fear of acceptance and/or retaliation, have been shown to have a lower publication rate over time compared to those LGBT+ individuals who do disclose (29).

Fortunately, professional oncology societies are recognizing the need and benefits for diversification of the workforce and its downstream effects on patient care and research advancements (21, 30, 31). The American Society of Clinical Oncology (ASCO) is focused on enhancing diversification of the workforce with the intent to lessen cancer disparities and improve poor trial enrollment by patients from underrepresented groups (32). The Society of Neuro-Oncology (SNO) found that its non-White members reported higher rates of unconscious biases/microaggression, inclusion disparities, and a lack of mentorship (33). Mentors acting equitably and inclusively will help these societies meet their goals.

However, despite efforts to enhance diversification within oncology, improvements within the pediatric oncology field are needed – in particular, there is still limited ethnic diversity within the workforce (14, 15). Recently, only 5–10% of the workforce identified as African American or Hispanic, with similar low leadership statistics: <10% identifying as African American, Hispanic, or Native American. Between the lack of clinician diversity and the low diversity in clinical trial enrollment, it begs the question on the level of advancement that is truly being made identifying genetic predispositions, optimal treatments, pharmacology of disease and therapies, and optimizing clinical care (34, 35).

The COVID-19 pandemic led to increased use of virtual communication technologies and coincidentally provided a real-world demonstration of the value of increasing diverse representation. Easier establishment of collaborations both national and international in scope permitted greater cross-institutional mentorship opportunities (36). The use of virtual meetings enhanced academic productivity, mentorship, networking and programming for pediatric hematologists and oncologists alike (36).

### Equity

The above converges on the following message: mentors have both concrete (e.g. enhanced productivity and mentee retention) and more conceptual (e.g. future workforce) reasons to tailor their mentoring to support diverse mentees. This is particularly relevant given recent court decisions, such as the rulings overturning affirmative action and permitting the denial of services for LGBT+ individuals. These actions have resulted in members of historically marginalized and disenfranchised groups feeling vulnerable. This in turn presents the risk of mentors and mentees finding themselves on uncertain ground. Mentors can and should work proactively to support their mentees by consciously striving to support them in an equitable fashion. Promoting scientific communities rooted in psychological safety enables mentees to perform at their best, building self-confidence and efficacy (37).

### Justice

Organizational justice reflects distribution of resources, support, and decision making and outcomes. Organizational justice is defined by an employee’s perception of the equity of their workplace. This is linked to emotional well-being and productivity within the workplace. In oncology specifically, supervisors with transformational leadership styles were associated with high organizational justice, translating to a positive quality of work life (QWL) (38, 39). Transformational leadership includes seven items: a vision, staff development, supportive leadership, empowerment, innovative thinking, leading by example, and charismatic leadership (40). Investigations of organizational justice and inclusion within pediatric oncology are limited, and more studies are warranted to define their relationship to workplace well-being and productiveness for both mentees and mentors.

In mentoring, the overarching concept of justice can be realized by having attending physicians and other faculty members work actively to support fellows, residents, medical students, and other trainees – both those they work directly and frequently with, and those who they encounter and work with for shorter periods. The relevant skills include those for *intervention*, *allyship*, and more *general strategies* (**Figure 1B**). It is imperative for mentors to create deliberate avenues and safe spaces for communication. A mentee feeling comfortable to express concern about a deadline or that personal obligations do not allow them to stay late at work are examples of such communication. Consciously encouraging a mentee’s wellbeing assists with their identification of supportive needs for professional development and can in turn promote their productivity – benefiting both mentee and mentor (41).

## DEI-based skills and examples

Provided below are relevant skills for mentoring in an inclusive manner, as well as example scenarios. These represent actions that can be undertaken by individual mentors, not the sort of organization or systemic interventions and changes that are beyond the scope of this piece. It is also important to note that while these examples demonstrate mentors being supportive of issues that impede productivity, the reality is that URM individuals are subject to biased assessments of their productivity: there are long-standing gaps at the level of academic promotion, tenure decisions, and NIH-sponsored research awards for minority faculty (42–44). In the case of tenure decisions this persists despite attempts to address this gap such as increasing tenure clocks and clock ‘extensions’ (45). A singular mentor can do their best to support their mentee(s) and be an advocate for change, but change also needs to come at organizational levels.

Skills are based on establishing a meaningful mentor-mentee relationship as the foundation, and for mentors to actively educate themselves and seek out understanding of mentee identities. Beyond role modeling inclusive behavior, inclusive mentoring requires establishing trust, placing the onus on the mentor given the power differential between mentors and mentees. Mentors must become educated about their mentees and trainees, including asking about preferred pronouns and understanding cultural events and holiday observations. Developing an awareness of mentees is critical as such information provides context to how individuals work optimally, how they prefer to receive feedback, and how people prefer to be recognized. Mentor-mentee relationships can also be structured in more formal ways, such as with an individual development plan (IDP) (46, 47).

### Microintervention

**Figure 2:**
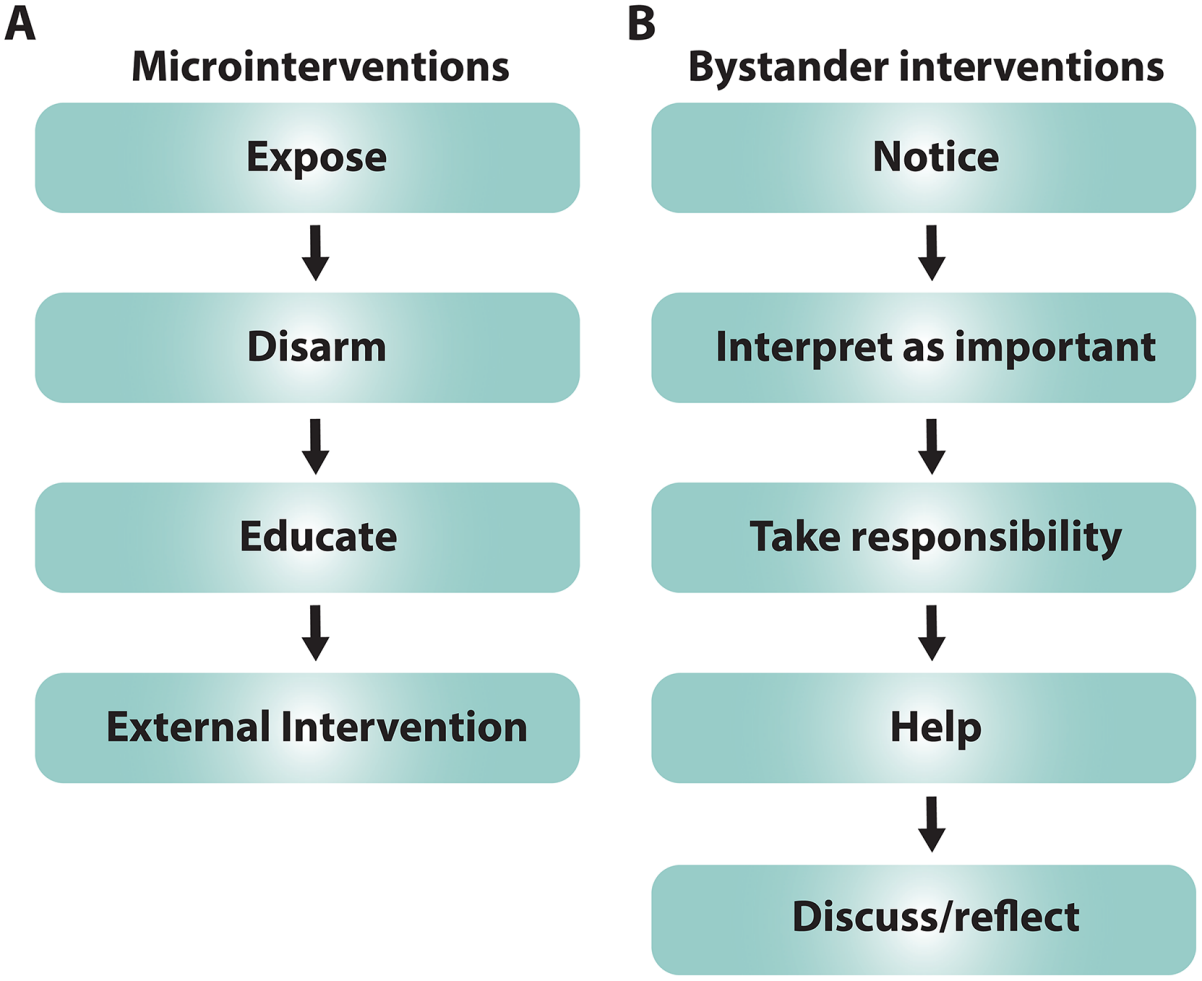
Intervention skills. **(A)** The approach to *microinterventions* depicted as a step-by-step process. **(B)** As in A, the approach to *bystander interventions* is described step-by-step.

Microintervention (48) is a four part process taking place in the moment: first exposing the situation, then disarming it before educating those involved, while intervening to help improve the situation (**Figure 2A**). Microintervention is analogous to ‘teaching in the moment’ that often occurs in clinical contexts – a skill that improves with practice.

Example: Christina is a first-year fellow. In the clinic workroom, you overhear attending physicians complaining that she is taking too long to see patients. You are also an attending and have previously met with Christina and know that she is away from her entire family, as well as her partner. You interrupt [Expose] the attendings and ask them how their clinic days are going [Disarm]. In conversation, you remind them that first year fellowship is extremely busy and taxing [Educate], and that staff should support Christina to get her work done efficiently [Intervention 1]. In addition, any constructive feedback to give to Christina to improve her workflow should be provided directly [Intervention 2].

### Bystander intervention

Bystander intervention, like microintervention, requires a mentor to realize that an issue is occurring and help address it, but unlike a microintervention, it *may* involve effort over time (49) (**Figure 2B**). The mentor must notice an issue, realize that it is important, take responsibility for addressing it, provide help, and discuss the issue.

Example: You are on a scholarly oversight committee for a hematology-oncology fellow, John. John is progressing well in his research, but you know that he and his family feel impacted by recent SCOTUS decisions. He has confided in you about this, and that he feels distracted recently. During the meeting, another committee members states that he needs to “focus more if he wants a career in academia” [Notice]. John is visibly upset, and you immediately intervene [Interpret as emergency/important] to state that, in your view, he is doing his best to balance his responsibilities at work and at home. After the committee meeting, you speak to John directly [Take responsibility] and you also arrange for a time to speak to the other committee member [Help, Discuss, Reflect].

### Allyship

Allyship is rooted in self-awareness with a focus on the platinum rule - treating others the way they wish to be treated, not as you wish to be treated (50). Another important consideration is self-education. Strong allies (and mentors) educate themselves on relevant topics rather than relying on the labor of others to become educated.

Example: You are one of the faculty interviewers for a program at your institution. During introductions, one of the candidates expresses their preferred pronouns are “they/them.” One of the Program Directors repeatedly refers to this person as “him” instead of “them” despite the clear expressed preference for an entirely different pronoun [Platinum rule]. You pull the Program Director aside and remind them to respect the preferences of others and to make an effort to utilize the correct pronouns [encouraging them to be Self-aware and Self-educate]. You make a note that for future interviews, you will ask candidates for their pronouns prior, and ensure that all faculty interviewers have this information [Help, Discuss, Reflect].

### General strategies and scenarios

Finally, there are strategies and skills that are rooted in general communication approaches between mentor and mentee. Techniques like good listening skills based in understanding first before giving advice or feedback, and being real and open with mentees will help meet them where they are in the moment, rather than where you imagine they should be, therefore providing them space to express themselves more fully. And while sponsorship is distinct from mentorship, acting as a sponsor for mentees by providing them with new opportunities they would not otherwise have available is another mechanism to support mentees.

Example: You are the principal investigator of a laboratory, and a fellow works in your lab. In the last month she has been absent from lab meetings, not performing key experiments, and overall less engaged [Notice]. She is typically enthusiastic and helpful with other lab members’ projects. At her next one-on-one meeting, you ask her how she is doing, and she replies “Not great.” You ask her to expand upon this, but only if she wants to discuss more, so that you can understand how to provide her with support [Interpret as important]. She is appreciative of this and explains that four of her patients have relapsed recently, and this has been difficult to manage with her research obligations. She tells you that she feels overwhelmed and feels such a responsibility for her patients’ care. You tell her you appreciate her sharing with you, and acknowledge how difficult it is to manage ill patients while balancing research and your own emotional state. You share that you too still find difficulty in balancing research with the care of ill patients [Be authentic and open to vulnerability]. You provide her with the contact information for employee wellness and ask if it is ok for you to share this information with her fellowship director for additional support [Educate and Intervene].

## Special consideration: the mentor as under-represented minority

The minority tax is a phenomenon whereby underrepresented individuals take on extra responsibilities for diversity and equity efforts. This has also been termed the “underrepresented minority in medicine faculty responsibility disparity” (51). These individuals also routinely face racism, sexism, exclusion (52, 53), lack of mentorship, and lower rates of promotion (43, 54, 55). The time advancing equity and inclusion is often not viewed favorably in the promotion process and can sway faculty from essential activities (56). Addressing the minority tax on mentors is ultimately the responsibility of institutions. Institutional systemic changes that distribute DEI efforts across faculty will assist in offsetting this tax on underrepresented individuals (57–60). Allyship and the above skills providing inclusivity help to alleviate the onus on URM individuals and reduce inequities in the workplace.

## Discussion

Mentoring and the mentor-mentee relationship are critical to the development of pediatric oncologists, in addition to clinicians and scientists broadly. The skills above are not restricted to the training and development of pediatric oncologists, but can and should be used in varied settings. All mentees deserve an environment that permits them to flourish. Mentees do not exist in a vacuum. They are individuals with rich and complex lives outside of the work environment that in turns influences their work.

Additionally, concepts like the platinum rule and striving to practice good communication skills are not just integral to mentoring, but to patient care. Pediatric oncology patients and their families are in a tremendously vulnerable position, and deserve care that reflects the same sensitivity to the experience of others that is present in inclusive mentoring. Developing and practicing such skills therefore advances not only mentorship goals, but clinical care.

Ultimately, it is imperative that mentors consider mentees’ identities in their mentoring behavior and strive to be inclusive. Successful mentoring is rooted in trust and enables a mentee to thrive. For mentees from underrepresented groups, that means not only that mentors should be allies and intervene in problematic situations, but that mentors should 1) seek to educate themselves about mentees’ identities, 2) encourage and promote the career trajectory of such mentees, 3) seek out opportunities to be sponsors and 4) invite mentees to be a voice at the table. In the end, it is not only the just thing to do for individual mentees, but the best thing to do for both the field of oncology and the patients for whom we care.

## Data Availability

The original contributions presented in the study are included in the article/supplementary material. Further inquiries can be directed to the corresponding author.
